# Concussion with primary impact to the chest and the potential role of neck tension

**DOI:** 10.1136/bmjsem-2018-000362

**Published:** 2018-10-16

**Authors:** Ron Jadischke, David C Viano, Joe McCarthy, Albert I King

**Affiliations:** 1 McCarthy Engineering, Windsor, Ontario, Canada; 2 Department of Biomedical Engineering, Wayne State University, Detroit, Michigan, USA; 3 ProBiomechanics, Bloomfield Hills, Michigan, USA

**Keywords:** football, upper neck forces, Hybrid III dummy, concussion, mTBI, brainstem

## Abstract

**Objectives:**

Most biomechanical research on brain injury focuses on direct blows to the head. There are a few older studies that indicate craniocervical stretch could be a factor in concussion by causing strain in the upper spinal cord and brainstem. The objectives of this study are to assess the biomechanical response and estimate the strain in the upper cervical spine and brainstem from primary impact to the chest in American football.

**Methods:**

Impact testing was conducted to the chest of a stationary unhelmeted and helmeted anthropomorphic test device (ATD) as well as the laboratory reconstruction of two NFL game collisions resulting in concussion. A finite element (FE) study was also conducted to estimate the elongation of the cervical spine under tensile and flexion loading conditions.

**Results:**

The helmeted ATD had a 40% (t=9.84, p<0.001) increase in neck tensile force and an 8% (t=7.267, p<0.001) increase in neck flexion angle when compared with an unhelmeted ATD. The case studies indicated that the neck tension in the injured players exceeded tolerable levels from volunteer studies. The neck tension was combined with flexion of the head relative to the torso. The FE analysis, combined with a spinal cord coupling ratio, estimated that the strain along the axis of the upper cervical spinal cord and brainstem was 10%–20% for the combined flexion and tension loading in the two cases presented.

**Conclusion:**

Strain in the upper spinal cord and brainstem from neck tension is a factor in concussion.

**What are the new findings?**

- There has not been much recent focus on the potential importance of neck tension causing concussion.
- The mass of a helmet added to a head can result in increased neck tension forces in impacts primarily to the chest.
- Compared with direct helmet-to-helmet collisions causing concussion, these impacts primarily to the chest result in lower head accelerations and angular velocities.
- Neck tension or strain along the axis of the upper cervical spinal cord and brainstem is a possible mechanism of brain injury and should be considered in the design and evaluation of helmets.

**How might it impact on clinical practice in the near future?**

- These findings could help identify a mechanism of concussion in sport.
- These data could be used by helmet manufacturers to develop protective equipment to reduce the incidence of concussion in sport and also methods to treat injured athletes.

## Background

The 2012 consensus statement on concussion in sport included the statement that ‘*concussion may be caused by a direct blow to the head, face, neck or elsewhere on the body with an impulsive force transmitted to the head.*’^1^ There are few studies on concussion with primary impact to the chest and the study of this type of collision may shed some light on a mechanism of injury.

In animal testing, Friede^2 3^ studied the mechanics of concussion by evaluating the signs and neuropathology in the upper spinal cord and brainstem of cats in response to a distraction load in a non-impact condition. He concluded that craniocervical distraction (tension) and flexion are the most important factors in concussion. Ommaya *et al* 4 produced signs of cerebral concussion, haemorrhages on and contusions over the surface of the brain and upper cervical cord by rotational displacement of the head on the neck, without direct head impact. They concluded that multiple mechanisms are involved in cerebral concussion, among them are rotational acceleration of the head, flexion-extension-tension of the neck and intracranial pressure gradients. Hodgson^5^ concluded that relative movement at the craniocervical junction may be an important factor in whether there is loss of consciousness in impacts resulting in inertial loading of the head.

In the human, sled testing conducted by Col John P Stapp^6^ resulted in the loss of consciousness of one volunteer at a peak sled deceleration of 38 g with an onset rate of 1370 g/s without impact to the head. Hutchinson^7^ conducted a video analysis of 174 concussion-causing hits in the NHL. Twenty per cent of these injuries had a primary shoulder-to-chest contact, but less than 5% had no secondary head contact. King *et al* 8 used a discrete parameter model of the head and neck to study the response of the neck of pilots who ditch in the ocean and fail to eject before the jet aircraft sank. Results showed that, with the added weight of a helmet, one of the reasons for the pilots failing to eject was cord concussion due in part to upper cervical cord stretch during the combined vertical acceleration and forward deceleration of the aircraft. The computed head linear and angular accelerations were below concussive levels. Ommaya,^9^ Hodgson^5^ and Jadischke *et al* 10 also indicated that the mass of the helmet aggravates the potential for injury by adding bending, axial and shear loads at the craniocervical junction.

The objective of this study was to assess the biomechanical responses from impact to the chest in American football. This study was completed in three phases. First, impact testing was conducted to the chest of a stationary anthropomorphic test device (ATD), both helmeted and unhelmeted. Second, a case study of two NFL game collisions was conducted to estimate the biomechanical forces in real-life collisions resulting in concussion. In these cases, the primary impact was to the chest, and the player experienced a concussion with a delayed return to play. Third, a finite element (FE) study was conducted using the head and neck from the Global Human Body Model Consortium (GHBMC) Average Male model to estimate the strain along the axis of the cervical spinal cord and brainstem under combined tensile and flexion loading conditions.

## Materials and methods

### Test series 1: general impact testing

Impact tests were conducted with head, neck and upper torso of a Hybrid III 50th percentile ATD struck at the centre of gravity of the chest. The 14 kg pelvis of the ATD was replaced with a 13 kg steel base. The ATD lumbar spine was vertical and the ATD was placed on a height-adjustable table. The tests were conducted by striking the stationary ATD with a 45 kg impactor with a 38.1 mm (1.5 inch) thick deformable vinyl nitrile end cap at impact speeds of 5–10 m/s. This end cap is used commonly in helmet-to-helmet testing to simulate a helmeted player.^11^ The impacts were repeated back-to-back with the ATD helmeted and unhelmeted. In the 9 and 10 m/s impacts, the facemask was removed to prevent it from striking the impactor ram. Details regarding the ATD instrumentation and filtering are found in the online supplementary figure S1. The ratio of the biomechanical responses from the ATD in the helmeted condition versus the unhelmeted condition was compared using a one-sided Student’s t-test.

10.1136/bmjsem-2018-000362.supp1 Supplementary data

### Test series 2: laboratory reconstructions

Game video was analysed to assess the heading angles, torso angles and closing speeds of two cases in the NFL with primary impact to the chest that resulted in concussion. The independent analyses from multiple camera views resulted in the estimated helmet location overlaying each other when plotted in three-dimensional (3D) model of the playing field. The scaled model of the playing field, distance travelled by the player’s helmet and the time between frames were used to estimate the preimpact speed and heading angle of each of the players. The players’ speeds were also checked using a two-dimensional analysis of the markings on the playing field. The helmet delta-V was calculated graphically (vector subtraction) using the average speed and 3D heading angle for 0.1 s prior to impact and 0.1 s after impact.

In the laboratory, the upper bodies of two Hybrid III 50th percentile ATDs were used to represent the football players involved in these collisions. The ATDs consisted of the Hybrid III head, neck, upper torso, shoulders, standing lumbar spine and pelvis and were ballasted using a weight vest to represent the player’s upper body mass. A nylon stocking was placed over the Hybrid III headforms to reduce the friction at the head-helmet interface and to provide a more realistic response of the helmet on the headform. This is consistent with NFL helmet testing.^12 13^ A large-sized American football helmet weighing 2.15 kg was fitted onto the ATD headform representing the player struck in the chest, and a large-sized American football helmet weighing 1.85 kg was fitted onto the striking ATD headform. The brow pads were positioned 2.54 cm (1 inch) above the top of the nose. The chin strap was attached so that it fit snugly over the Hybrid III chin. Data acquisition and instrumentation for each of the ATDs were similar to that described in test series 1. Additional information is provided in the online supplementary figures S2 and S3.

### FE modelling

The head and neck were segmented from the whole GHBMC 50th percentile male model at the first thoracic vertebra along with all relevant musculature and ligaments. Validation of the head and neck was previously completed by others^14 15^ using cadaveric and volunteer experimental data. In the present study, the model was not used to assess tissue-level strains in the brainstem and spinal cord directly because there was no specific validation related to the brainstem and upper cervical spinal cord discussed in the literature. Rather, the kinematics of the vertebrae and skull were studied to assess the craniocervical stretch in the vertebral column in response to independently applied tensile (distraction) loading and forward flexion. These were the primary biomechanical responses of players in these impacts to the chest. The elongation of the cervical column was measured using nodes defined on the anterior, left, right and posterior sides of each cervical vertebra, and the location and orientation of the skull was monitored by tracking its centre of gravity.

The average strain in the cervical spine was assessed at the level of C1–C5 since the literature^16 17^ has shown there to be caudal (downward) displacement of the spinal cord relative to the spinal column in this level and cephalad (upward) displacement of the spinal cord below this level indicating that stretch of the spinal cord (above C5) and brainstem occurs. A spinal cord coupling ratio of 0.65^18 19^ was applied to the vertebral column strain to estimate the strain along the axis of the spinal cord and brainstem relative to vertebral body strain. The kinematics predicted by the FE simulations were compared with existing human volunteer^17 20^ and cadaveric studies.^21 22^ Additional information is provided in the online supplementary figure S3.

10.1136/bmjsem-2018-000362.supp3 Supplementary data

## Results

### Test series 1: general impact testing

**Figure 1:**
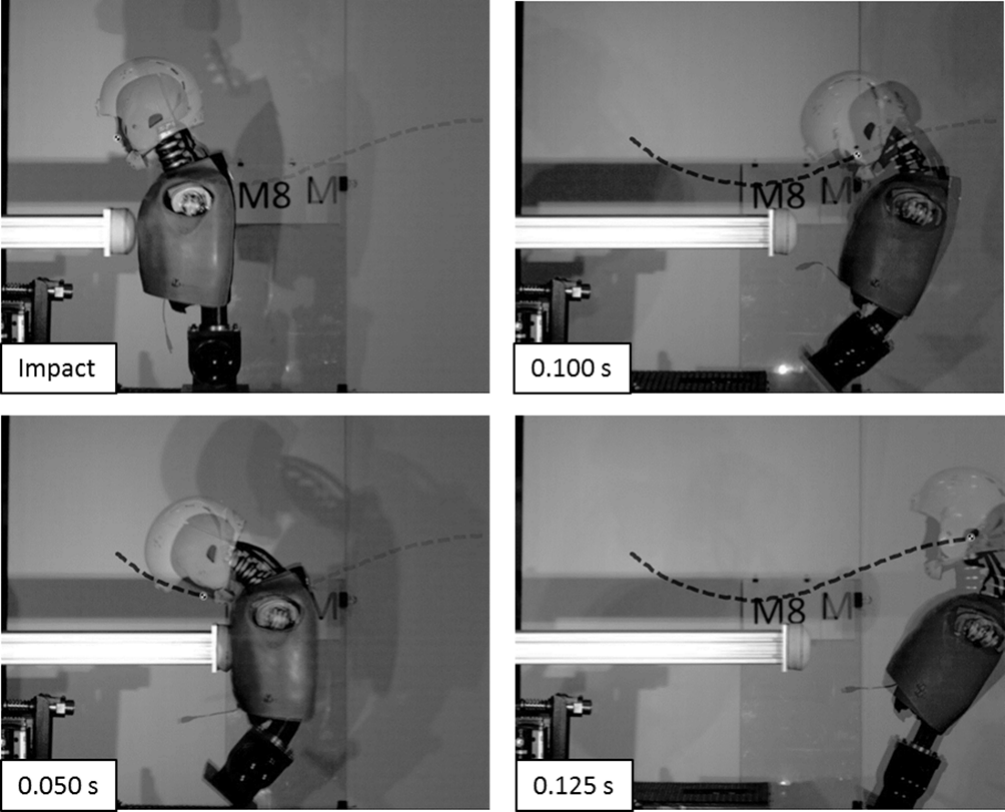
Comparison of a helmeted versus unhelmeted chest impact. The unhelmeted impact is overlaid onto the helmeted impact.

The primary ATD response to chest impact was in the sagittal plane. Table 1 illustrates the biomechanical responses for various closing velocities. There was a 40% ± 10% (t=9.84, p<0.001) increase in upper neck tensile forces when compared with unhelmeted impacts of equal severity. There was also an increase of 8% ± 3% (t=7.267, p<0.001) in head flexion angle. There was a reduction in head displacement of 18% ± 4% and a reduction of rotational velocity of 18% ± 6%. The head motion lagged behind the torso motion to a greater extent in the helmeted impacts. The helmet mass (2.15 kg) increased the effective mass of the headform (4.54 kg +2.15 kg) by 47% when compared with the unhelmeted headform (4.54 kg). This resulted in significantly greater neck forces and moments when compared with the unhelmeted impacts. High-speed video of a 10 m/s chest impact is illustrated in figure 1. Additional information is provided in the online supplementary video 1.

10.1136/bmjsem-2018-000362.supp5 Supplementary video

**Table 1:** Summary of ATD data from test series 1

Test ID	Helmet	Impact	Impact	Head kinematics							Chest	Upper neck		
				Translation					Rotation		Acceleration	Forces		Moment
		Speed	Force	Acceleration	Velocity		Displacement		Velocity	Rotation				
		(m/s)	(N)	resultant	x	z	x	z	y	y	res	Shear	Tension	Flexion
				(g)	(m/s)	(m/s)	(m)	(m)	(rad/s)	(°)	(g)	(N)	(N)	(Nm)
M8B	No	5	4187	12.3	−6.19	3.11	−0.34	0.20	−13.9	−24.8	10.7	−326	387	15.0
M8A	Yes	5	4026	11.1	−4.73	2.62	−0.27	0.20	−12.3	−25.7	10.0	−421	522	16.0
M8C	No	6	4931	15.1	−7.36	3.81	−0.40	0.24	−17.8	−30.5	12.9	−384	484	17.7
M8D	Yes	6	4931	13.7	−6.16	3.21	−0.32	0.24	−15.6	−33.1	12.5	−481	676	19.0
M8F	No	7	5736	17.9	−8.20	4.92	−0.44	0.31	−20.9	−34.8	15.3	−448	602	19.4
M8E	Yes	7	5655	16.1	−6.96	4.14	−0.36	0.30	−16.4	−38.0	14.8	−587	868	19.8
M8G	No	8	6681	21.2	−9.46	6.01	−0.50	0.37	−25.2	−40.1	18.0	−533	696	22.0
M8H	Yes	8	6621	19.2	−7.60	4.74	−0.39	0.37	−18.5	−42.5	17.8	−682	1094	22.3
M8K	No	9	8029	25.7	−10.33	7.60	−0.56	0.47	−29.3	−44.5	21.4	−650	868	23.8
M8J	Yes*	9	8130	25.2	−8.94	6.35	−0.48	0.44	−23.4	−48.6	21.0	−747	1195	26.9
M8L	No	10	11 449	35.6	−11.44	9.04	−0.63	0.55	−36.0	−48.2	31.2	−816	1322	34.7
M8M	Yes*	10	11 771	37.2	−10.16	7.29	−0.55	0.50	−29.3	−53.7	32.1	−955	1687	39.7
Average (helmet/unhelmeted)				0.94	0.83	0.83	0.82	0.97	0.82	1.08	0.98	1.24	1.40	1.07
SD (helmet/unhelmeted)				0.06	0.04	0.02	0.04	0.03	0.06	0.03	0.03	0.07	0.10	0.06
t				2.495	9.040	18.733	12.110	2.031	7.817	−7.177	1.657	−8.900	−9.839	−3.322
P (one tail, 0.05)				0.027	<0.001	<0.001	<0.001	0.049	<0.001	<0.001	0.079	<0.001	<0.001	<0.001

ATD, anthropomorphic test device.

*Facemask removed to prevent contact with impactor ram.

### Test series 2: laboratory reconstructions

**Figure 2:**
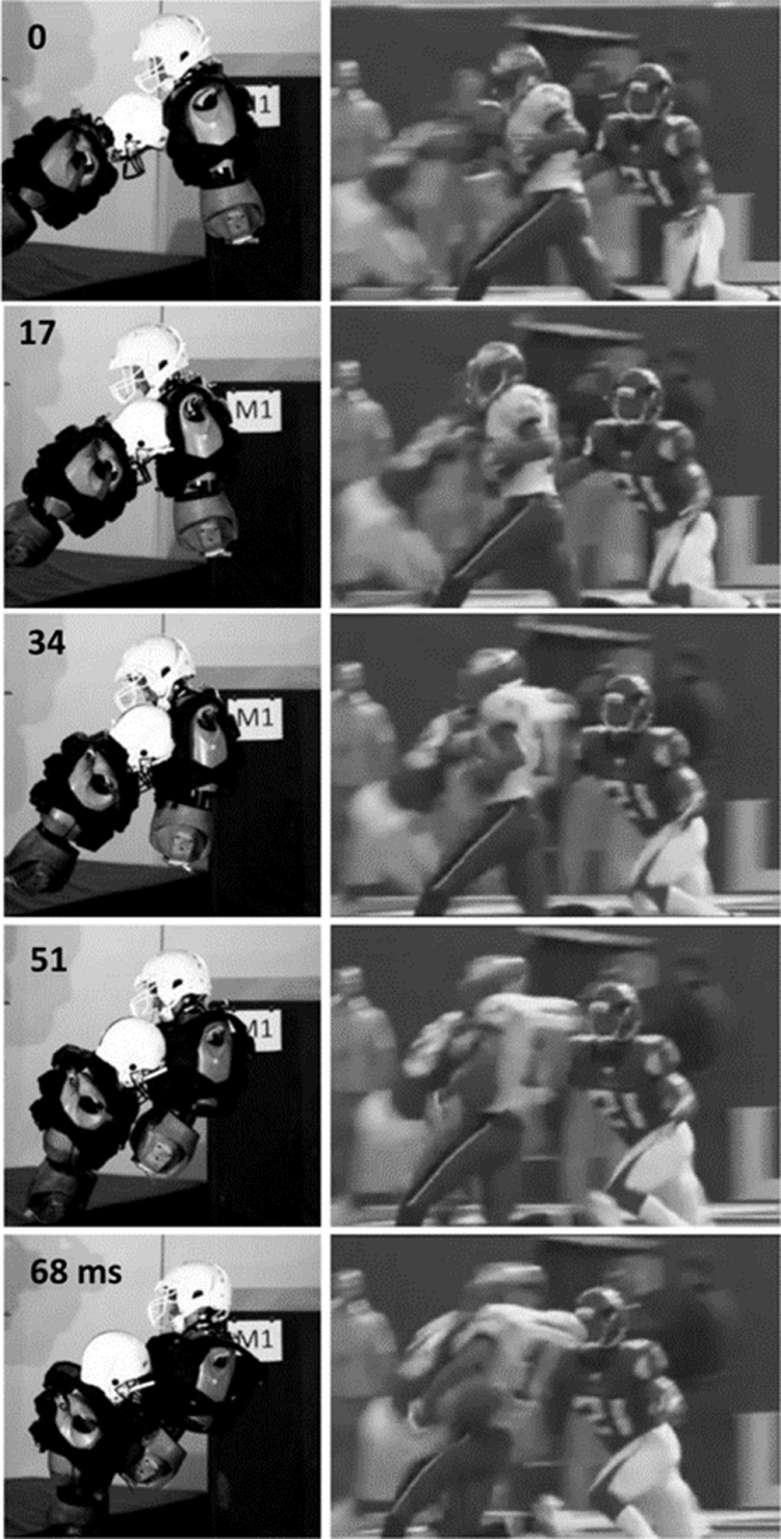
Comparison of the game impact for case A.

**Figure 3:**
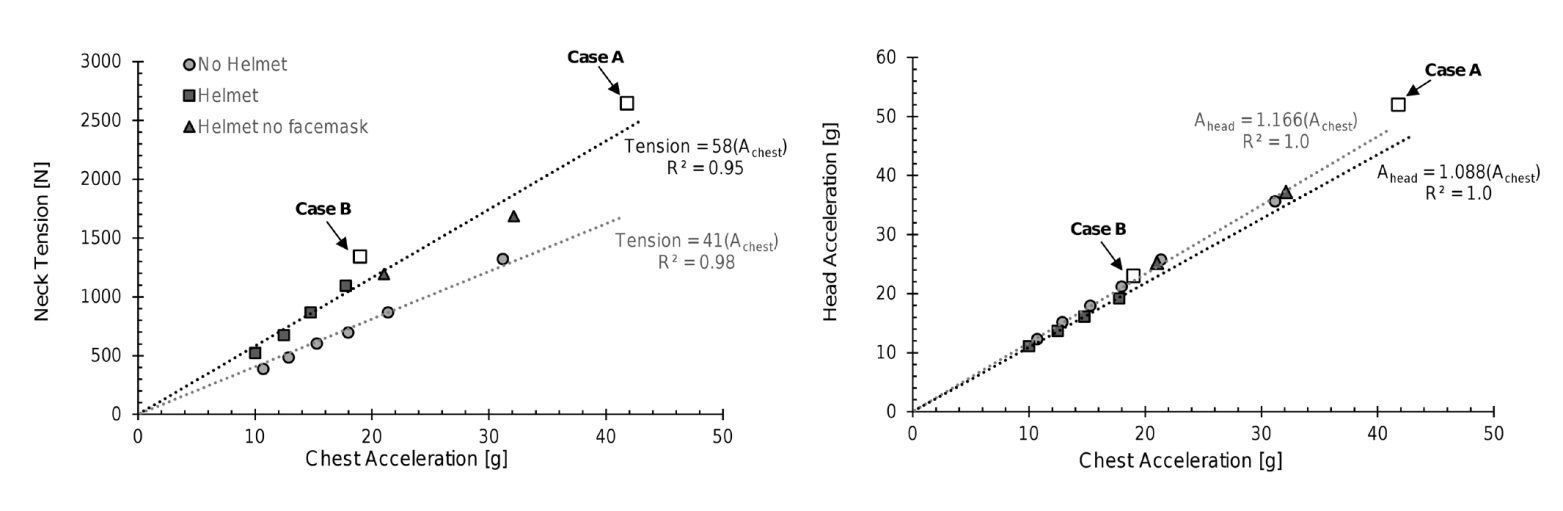
Neck tension and head acceleration versus chest acceleration for test series 1 and the laboratory reconstruction of case A and case B.

The closing velocities for case A and case B were 12.6 and 9.8 m/s, respectively. The reconstruction data from the struck ATD are summarised in table 2. A comparison of the postimpact kinematics of case A is illustrated in figure 2. A comparison of these laboratory reconstructions to the test series 1 results for the helmeted and unhelmeted ATDs is illustrated in figure 3.

**Table 2:** Summary of ATD data representing the struck and injured player from the laboratory reconstructions

Case	Closing	Location	Kinematics									Upper neck kinetics		
	Speed		Translational acceleration			Translational Δvelocity		Translational displacement		Rotational velocity	Rotation	Forces		Moment
	**(m/s)**		**x**	**z**	**resultant**	**x**	**z**	**x**	**z**	**y**	**y**	**Shear**	**Tension**	**Flexion**
			**(g)**	**(g)**	**(g)**	**(m/s)**	**(m/s)**	**(m)**	**(m)**	**(rad/s)**	**(** **°** **)**	**(N)**	**(N)**	**(Nm**)
A	12.6	Head	−38.2	49.2	50.9	−12.20	7.89	−0.73	0.55	−41.1	−51.0	−1074	2646	49.3
		Chest	−36.6	18.0	41.8	5.1	1.6	−0.42	0.08	8.2	10.1			
B	9.8	Head	−15.6	−15.0	18.7	−6.10	−3.40	−0.16	−0.14	−26.5	−46.0	−799	1342	36.0
		Chest	−19.2	9.7	19.0	3.8	0.9	−0.17	0.03	4.6	3.0			

ATD, anthropomorphic test device.

### FE modelling

FE modelling indicated that the strain in the vertebral column increased linearly with head flexion or tensile loading; however, it varied along the length of the cervical spine. The average strain in the vertebral column in flexion was 0.21% strain/degree of head rotation and 4.6% strain/1000 N of tensile load. The maximum strain in the vertebral column was predicted to occur in the upper cervical spine (C1–C2) and was 0.28% strain/degree of head rotation and 6.5% strain/1000 N of tensile load for flexion and tension, respectively (table 3).

**Table 3:** Estimates of strain in the spinal canal (FE study) and CNS (FE study×0.65 spinal cord coupling ratio) for various neck tension loads and flexion angles of rotation. The estimates were applied to the laboratory reconstruction data to estimate the strain in the CNS of these injured players

Case	Tension					Flexion					Sum	
	Force	Average strain C1–C5		Maximum strain C1–C2		Rotation	Average strain C1–C5		Maximum strain C1–C2		Average strain C1–C5	Maximum strain C1–C2
	(N)	Spinal canal	CNS	Spinal canal	CNS		Spinal canal	CNS	Spinal canal	CNS	CNS	CNS
		(%)	(%)	(%)	(%)	(°)	(%)	(%)	(%)	(%)	(%)	(%)
FE study	500	2.4	1.6	2.1	1.3	35	7.4	4.8	9.8	6.4	–	–
FE study	1500	7.1	4.6	9.2	6.0	45	9.5	6.1	12.6	8.2	–	–
FE study	2500	10.5	6.8	16.9	11.0	55	11.6	7.5	15.4	10.0	–	–
Case A	2646	11.5	7.5	17.3	11.2	51	10.7	7.0	14.3	9.3	14.4	20.5
Case B	1342	5.9	3.8	8.7	5.7	46	9.7	6.3	12.9	8.4	10.1	14.1

CNS, central nervous system; FE, finite element.

A spinal cord coupling ratio of 0.65^18 19^ was used to estimate the central nervous system (CNS) strain relative to vertebral body strain. A maximum strain along the axis of the spinal cord and brainstem for a flexion angle of 55° was predicted to be 7.5%–10.0%. These estimates using FE modelling were comparable to in vivo volunteer data which measured a maximum strain in the spinal cord of approximately 10.2% at a 55° flexion angle.^17^ The average strain along the axis of the cervical spinal cord and brainstem was predicted to be 1.6%, 4.6% and 6.8% for neck tension loads of 500, 1500 and 2500 N, respectively. The peak strains in the upper cervical spine (C1–C2) were predicted to be 1.3%, 6.0% and 11.0%, respectively.

## Discussion

The laboratory reconstruction data for case A and case B, as well as the FE data, were used to estimate the strain along the axis of the spinal cord and brainstem in these concussed NFL players. The estimated strain was 13.0%–18.6% in case A and 8.7%–12.2% in case B due to combined tension and forward flexion. This range represents the estimated average strain (low) to the maximum strain (high). The estimated total strain accounts for the time-varying sum of the strains due to tension and flexion. The laboratory reconstruction and FE results indicate that the axonal strain in the spinal cord and brainstem (table 3) exceeds the levels that have been documented to cause changes in functional and structural response in spinal nerve roots when stretched in tension at varying strain rates.^23^ The strains are similar to those documented in in vivo tests with primates which resulted in functional changes in the spinal cord as well as changes in heart rate and respiration.^24^

While translational acceleration, rotational velocity and rotational acceleration of the head have been discussed as biomechanical correlates with concussion, craniocervical stretch resulting from tension and flexion in the upper cervical spine has also been reported to be an important factor in concussion.^2 3 5^ Neck tension and head flexion have each been shown to result in strain of the upper cervical spinal cord and the brainstem. In a study of 183 human cadavers, Breig^25^ found that tension generated in the spinal cord can be transmitted from the spinal cord to the brainstem. The largest elongation occurred in the medulla oblongata, and no elongation was apparent superior to the midbrain. The reticular formation of the brainstem controls heart rate, respiration and consciousness. The loss of consciousness in one of the players in this case study is consistent with injury to the brainstem.^2 3 5 24 26^

In case A and case B, the struck Hybrid III ATD underwent 51° and 46° of head flexion, respectively. The forward flexion of the head was combined with neck tension as a result of the inertial loading of the head and helmet. The flexion of the head is within normal range of motion of the human for quasistatic movement; however, in the human^17 18 27–29^ and primate,^16^ imaging studies have reported elongation of the cervical spinal canal and cord in flexion. The FE modelling results, combined with a coupling ratio, estimate strains in the CNS of 9.3% and 8.4% as a result of forward flexion, in cases A and B, respectively. These strains, by themselves, are within the range that has been documented for the human^17^ as part of the normal range of quasistatic flexion.

The neck tensions in this case study (case A=2646 N, case B=1342 N) are greater than the neck tensions found in volunteer studies^30–32^ and greater than uninjured NFL players^12^ (670±405 N). The neck tensions are similar to those reported by Viano *et al* 33 in their reconstruction of struck and injured players in the NFL (1704±432 N) and are less than the neck tensions resulting in failure of the cervical spine in musculoskeletal cadaveric studies.^21 22 34 35^ The tensile loads correspond to approximately 3.27 (case A) and 1.10 (case B) times the player’s body weight. This tensile load must be supported by the soft tissues of the neck. In these cases, the struck players did not appear to have the opportunity to ready themselves for the impact. From our FE study, and by applying a coupling ratio, the maximum strain in the CNS due to neck tension alone was estimated to be 11.2% and 5.7% for cases A and B, respectively.

The time-varying strain along the axis of the spinal cord and brainstem due to combined tension and flexion for case A and case B was on the order of 13.0% to 18.6% and 8.7% to 12.2%, respectively. The data presented in this case study support the mechanism of injury discussed by Friede^2 3^ and Hodgson and Thomas^36^ and Hodgson^5^ who have indicated that strains in the upper spinal cord and brainstem are important factors in concussion. The brainstem’s relation to concussion is further supported by the early work of Denny-Brown and Russell^26^ who produced concussion signs in the decerebrate animal.

The addition of the helmet to the ATD headform in test series 1 resulted in an increase in neck tension and forward flexion of the head. The neck tension increased by 40% and forward flexion increased by 8% as a result of the added helmet mass and inertia. Others^4 5^ have indicated that the mass of the helmet added to the head can increase the strain at the craniocervical junction. If, through further research, neck tension is found to be a biomechanical predictor of concussion, helmet and equipment manufacturers could use this information to optimise helmet performance and also to develop alternative methods of protecting against concussion.

There are several limitations of this study that should be noted. The case study is limited since only two cases were reconstructed. However, the reconstruction of these two cases may help shed some light on a potential mechanism of concussion since they investigated impacts that were primarily to the chest. This case study was performed using the Hybrid III ATD in a laboratory test environment. The Hybrid III headform and neck provide a biofidelic response in the loading condition analysed; however, it is not human, therefore tissue-level strains could not be directly assessed. The data acquired were used in conjunction with FE modelling to estimate the stretch in the upper cervical spine and a coupling ratio was applied to assess the strain in the CNS under these loading conditions. There are limited data that discuss spinal cord coupling ratio. However, the relative length of the spinal cord and brainstem when compared with C1–C5 also supports a coupling ratio of approximately 0.65 (online supplementary figure S4).

10.1136/bmjsem-2018-000362.supp4 Supplementary data

In case A, on impact, the torso’s forward motion stopped and the player’s head and helmet continued to move and flex forward. This indicates that the primary contact was to the chest of the struck player. Due to the severity of this collision, the bottom of the struck player’s facemask appears to have made contact with the top of the defending player’s helmet as his head flexed forward. This was also simulated in our laboratory reconstruction of the collision and appears to have reduced the forward flexion of the head and increased the neck tension in comparison to test series 1.

In this study, only strain in the neck has been considered from an impact to the chest. The rate of loading indicates the strain rate effect may be a factor in concussion and deserves further attention in the future. Additional limitations are discussed in the online supplementary video 1.

10.1136/bmjsem-2018-000362.supp2 Supplementary data
