# Supplementary figures and images for: Concussion with primary impact to the chest and the potential role of neck tension
Source: BMJ Open Sport Exerc Med. 2018 Oct 16;4(1):e000362. doi: 10.1136/bmjsem-2018-000362 (PMC6196936; doi:10.1136/bmjsem-2018-000362)

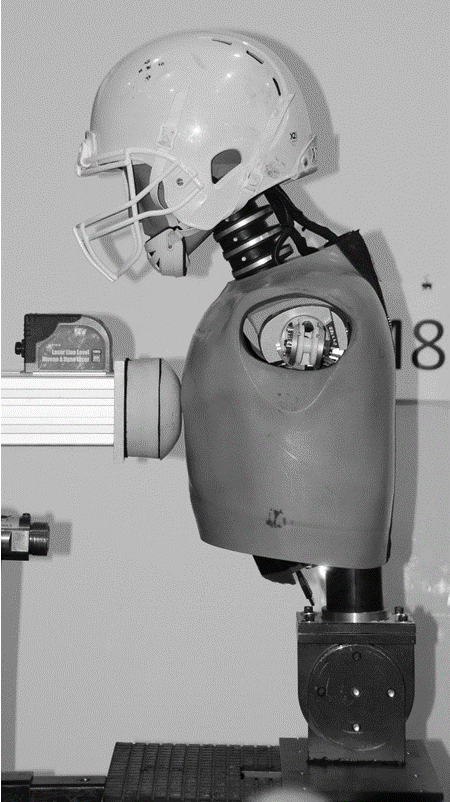

Supplement: Supplementary data [file bmjsem-2018-000362supp001.tif]

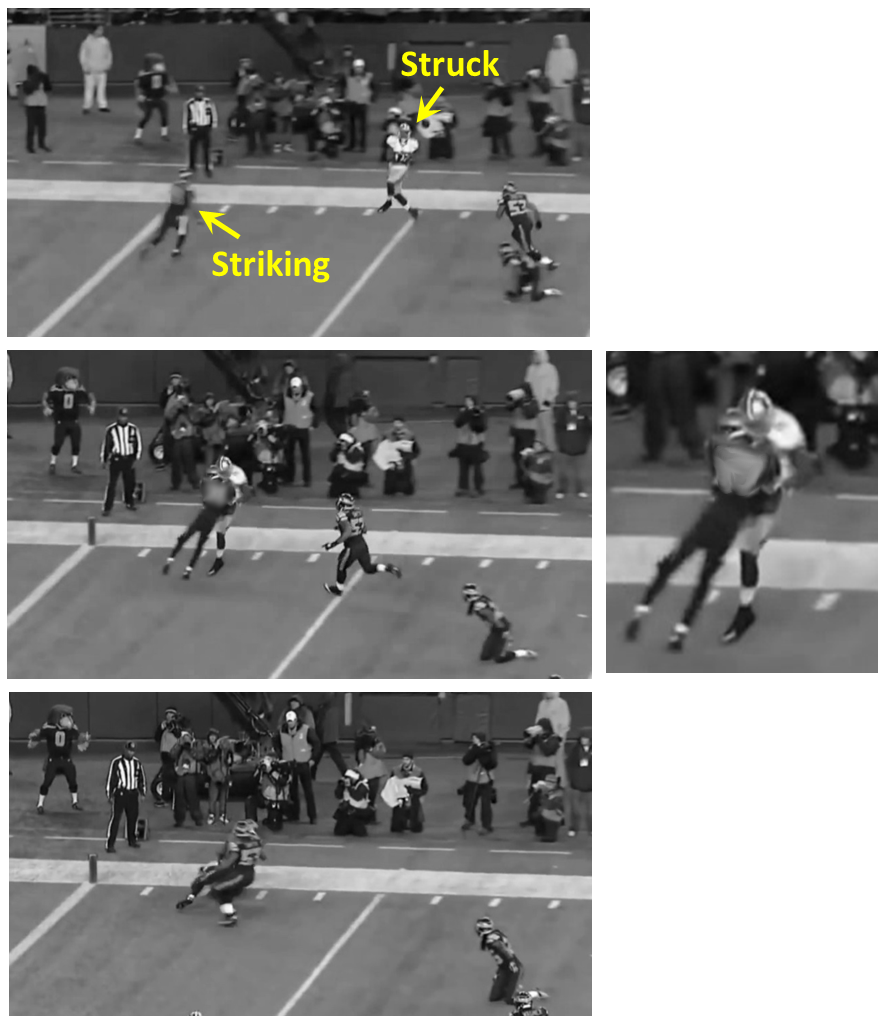

Supplement: Supplementary data [file bmjsem-2018-000362supp003.tif]

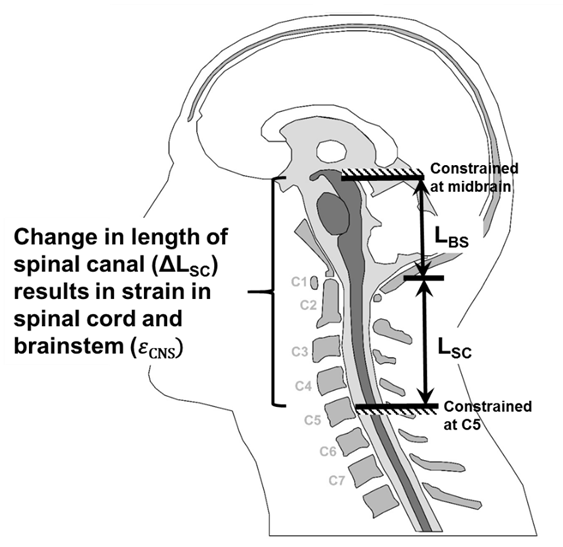

Supplement: Supplementary data [file bmjsem-2018-000362supp004.tif]

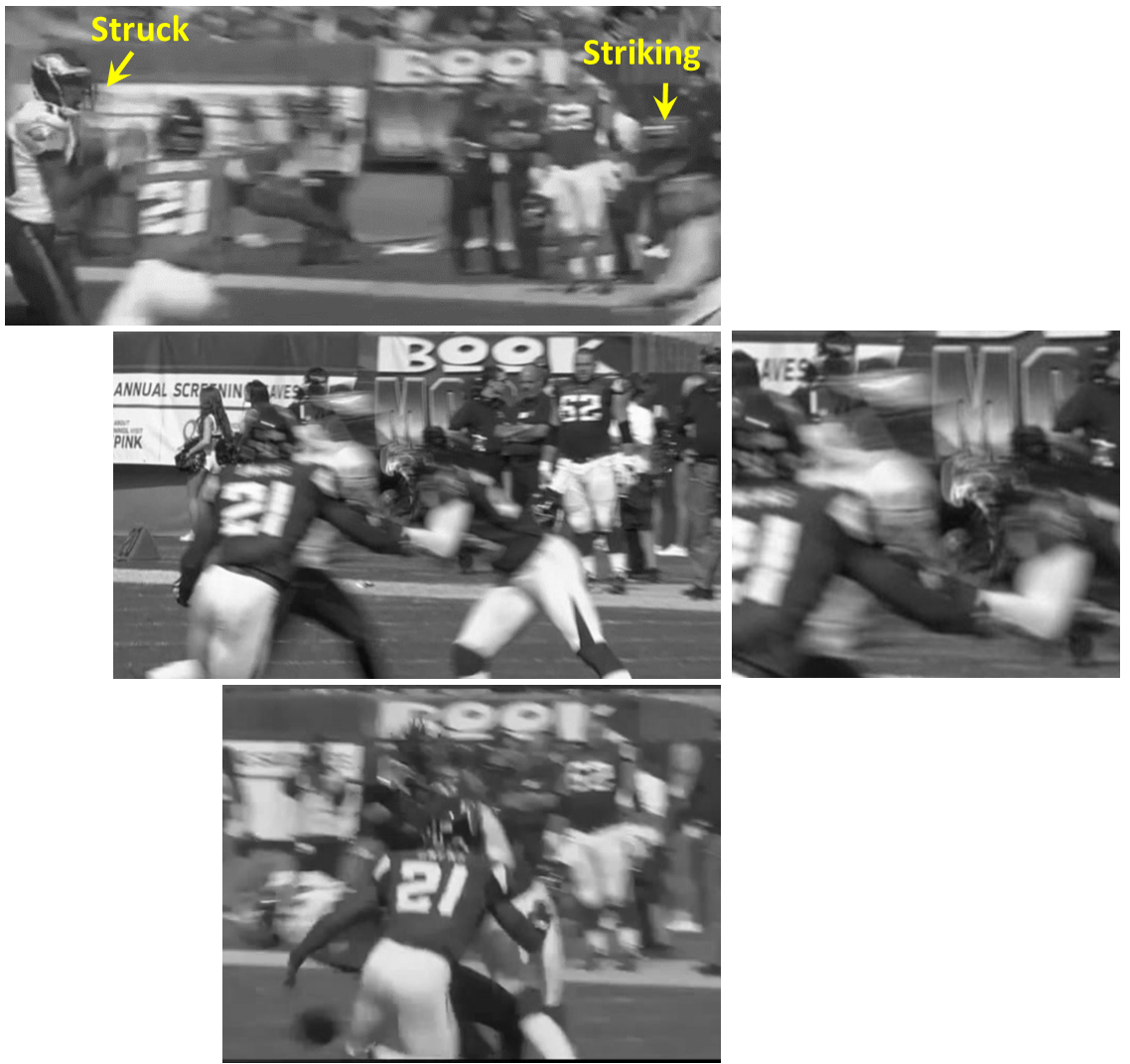

Supplement: Supplementary data [file bmjsem-2018-000362supp002.tif]
